# Salesmanship in Dentistry

**Published:** 1921-07

**Authors:** Herbert G. Frankel


### SALESMANSHIP IN DENTISTRY

BY DR. HERBERT G. FRANKEL

"Salesmanship is the ability to sell." What are some of the requirements of the successful salesman? As I see them, they are as follows:

1. Personality.
2. Nerve or grit.
3. A student of psychology.
4. Ability as a talker.
5. Ability to deliver the goods.
6. Ability to keep customers or patients after they are once sold.

I shall endeavor to bring before you the above topics and discuss each in turn.

### PERSONALITY

Personality, according to Webster, is "that which constitutes a person." I believe a better definition would be, "that trait in a person which either attracts or repels."

It means a great deal to your welfare if you have developed personality.

What constitutes personality and how is it developed?

The best way that I can explain this is to advise you to act natural. Have a pleasant word for everybody. Learn to think twice before you speak and then speak only good. Follow the maxim: "Hear no evil, Speak no evil, and Do no evil." *Smile.* We are all grouches, more or less and we should smile and forget our troubles. Be sympathetic, and be thoughtful, but do not be an "easy mark." Be reserved and firm when the occasion demands. Do not think that you can acquire personality without work, because it is impossible.

Why do you often say after meeting a man, "there is something about him that I like or dislike.'' It is his personality.

Youth or old age, make no difference, and a young man has just as much opportunity of developing personality as a man who is up in years.

I know a salesman who wins because he smiles, and he smiles his way into the hearts of his customers. They are glad to see him and look forward to his visits with pleasure.

Develop that latent faculty and it will help to bring the sunshine into your own life and the lives of others.

### NERVE OR GRIT

We usually say, that the fellow who can calmly light a cigarette, while looking into the business end of a loaded revolver, has, ''nerve.''

Again, if a fellow is in a game of football and gets a rib busted, but plays through the entire contest, we say, that is ''grit.''

What then is their connection with salesmanship?

Did you ever have a customer or patient, who in manner resembled a ''roaring lion'' and to approach them made the cold chills run down your spine? There are such individuals and we get them in the practice of a profession as well as a business, only they are at a disadvantage in a profession, as they seek you and you do not seek them. But, do not forget, you must sell them, just as the salesman would, were he to call on them.

Again, it takes nerve and grit for an individual to raise his prices and by salesmanship, sell his wares, in the face of a competitor, who lowers his prices.

You try to tell a patient that your price for a denture is so much and she or he will say, ''Well, I know where I can get it for less.'' Then is when you will have to show your nerve, grit and ability as a salesman, to prove to her that your article is superior and cheaper in the end.

The younger you are the more nerve and grit you will need and remember this, though ''the wolf be at the door''

and business is rotten, grin and bear it, because if its that way with you, the other guy is also in the same boat. Sometimes one feels as if he were a failure and then he realizes that he has just been fooling himself, as failure is due to the lack of backbone. A man with a spine like spaghetti will not go very far. You must keep a plugging and your watchword should be, "carry on." No matter how dark the clouds, there is always a silvery lining.

### PSYCHOLOGY

"Psychology is the science of the powers and functions of the soul."—Webster.

This has a great deal to do with salesmanship. Play upon your patient's mind until you find their weakness and it will show itself sooner or later and then harp on that subject.

If your patient be a golf enthusiast, talk golf and get their attention to such a degree, that when you do finally switch to the subject of selling them, you will have them enthused and interested. Now to do this, you must be able to talk on any subject and I shall speak of this later.

Take yourself for an example. You have some hobby and you like to ride it. Let us say that it is fishing. Now, you and I are introduced and in the course of conversation, you let it slip out that you are a fisherman, or perhaps I draw it out of you. Before we realize it we will launch upon a discussion, and we would finally part warm friends. So is the power of psychology which has to do with the power of the mind.

Learn to read the fellow, find out his hobbies and ride them with him as it will please him as well as yourself.

### ABILITY AS A TALKER

Small words well placed; short sentences used right; thought gems brilliantly orated, bring out the best in man. For example, "Lincoln's Gettysburg Address," "Mark

Anthony's Address,'' and Burke's ''Reconciliation'' are a few of the gems of our literature.

Cultivate good speech and learn to speak well. Read good books, topics of the day, current events, and above all cultivate the faculty of remembering names, faces and dates.

Avoid all subjects that you are not familiar with, but get familiar with the subject so you will not have to avoid it if it is ever brought up again. Touch upon subjects of a scientific nature as you can then easily switch to the Science of Dentistry. Read the history of your profession. Be able to tell your patients everything about dentistry.

Talk health. Your patients will be more than interested. They are anxious to know the whys and wherefores of health and especially when it has to do with their own well being. Speak of their diet. Show them the difference in diets of today and years gone by. Tell them why teeth decay and how it can be prevented. Explain to them how pyorrhea starts, how it can be prevented and the results if it is not taken care of in time.

If you are talking about appliances explain their bad points as well as their good points, but show the patient how the good points overcome the bad points. Draw diagrams, or if necessary, have models and show how the work is to be constructed. Sometimes you can convince by comparison. For example, I sold a gold denture, by letting the patient feel the difference in weight in an all vulcanite denture and one with a gold base.

Developed speech will bring success.

### DELIVER THE GOODS

Make an article that has merit, because it is an established fact, that an article must be something in order that it will be able to stand the storm of competition. You can not do everything perfect as no man is perfect, but you can give your best and by trying to produce better results, you improve your workmanship.

The best workman is the one who tries his best all the time, just as the best salesmen are those with the best goods. You can fool a man once, but not twice and if you do not do your best, you will not have repeaters.

I have seen trade marks which say, "We aim to please" or "You must be satisfied" and they strive to give the high grade merchandise.

If you were to buy an automobile tire guaranteed for 6,000 miles and after using it for 1,000 miles you were to have a blow out, you would want it replaced or else you would not go back to the place where you bought it.

We can not make guarantees in dentistry, but we can give service and by doing the best we know how, we can advertise ourselves and our business.

Give a thought to good work and strive for better dentistry. In this regards it behooves me to say a word about dental laboratories. Busy men can not afford to do their own work, hence the dental mechanic. Some of these men are excellent workmen, but a great number of them, have little conception of real dental work. Mechanical dentistry is an art and takes years of training, but all over the country, we have men posing as dental mechanics, who would not even make good helpers in a boiler factory. This condition though is being remedied by the establishment of schools for dental mechanics.

Good work is paramount in salesmanship.

### REPEATERS

How pleasing is the remark, "O, Doctor, that bridge you made for me some years ago, is as good as new. Please see if I need something else."

The real salesman is the one, who, after selling his patient or customer, can still resell him over and over again. Keep account of the number of times a patient returns. If you are having repeaters, your business is safe.

If Mrs. S. comes in and says that Mrs. B. sent her, and Mrs. B. is a satisfied patient, you are also safe. But if Mrs. B. comes in and then never comes in again, look out.

A patient dissatisfied is worse than a snake bite as the poison spreads and it hurts. Make your motto, "We aim to please" and go after repeaters and win. It takes time to get there and one must not be discouraged. The men at the top have as much trouble staying there as the fellow who is climbing and they must study and work too. Remember this that anyone can sell goods, by underbidding and substituting an inferior article, but the real salesman is the one who sells a good article at a fair price. Get as much as you are able to get for your art, but get good prices. The trouble with our profession is that we are not being paid just prices for our work. This can be accomplished by salesmanship and better business methods. Let us all get busy and establish these in our business and create a better and more profitable dentistry.

